# Sentiment Analysis Using Common-Sense and Context Information

**DOI:** 10.1155/2015/715730

**Published:** 2015-03-17

**Authors:** Basant Agarwal, Namita Mittal, Pooja Bansal, Sonal Garg

**Affiliations:** ^1^Department of Computer Science & Engineering, Swami Keshvanand Institute of Technology, Management & Gramothan (SKIT), Jaipur 302017, India; ^2^Department of Computer and Engineering, Malaviya National Institute of Technology (MNIT), Malviya Nagar, Jaipur 302017, India

## Abstract

Sentiment analysis research has been increasing tremendously in recent times due to the wide range of business and social applications. Sentiment analysis from unstructured natural language text has recently received considerable attention from the research community. In this paper, we propose a novel sentiment analysis model based on common-sense knowledge extracted from ConceptNet based ontology and context information. ConceptNet based ontology is used to determine the domain specific concepts which in turn produced the domain specific important features. Further, the polarities of the extracted concepts are determined using the contextual polarity lexicon which we developed by considering the context information of a word. Finally, semantic orientations of domain specific features of the review document are aggregated based on the importance of a feature with respect to the domain. The importance of the feature is determined by the depth of the feature in the ontology. Experimental results show the effectiveness of the proposed methods.

## 1. Introduction

The field of sentiment classification is an exciting new research direction due to the large number of real world applications where discovering people's opinion is important for better decision-making. Sentiment Analysis is the study that analyzes people's opinion and sentiment towards entities such as products, services in the text [1]. It has always been important to know what other people think. With the rapid growth of user-generated data on the Web, people are using online review sites, blogs, forums, social networking sites, and so forth for expressing their opinion. Therefore, a necessity of analyzing and understanding these online generated data/reviews has arisen. The user can know the merits and demerits of the product from the experiences shared by people on the web, which can be useful for them in decision-making. E-commerce companies can improve their product or services on the basis of people's opinion and current trends. The automatic analysis of online contents to extract opinion requires deep understanding of natural text by the machine; capabilities of most of the existing models are known to be unsatisfactory [2].

An opinion lexicon is a dictionary containing opinion words with their polarity value to indicate the positive or negative sentiments, for example, “happy,” “excellent,” “bad,” “boring,” and so forth. These opinion words are used in most of the existing sentiment analysis models as a key indicator of the opinion of the user. In the literature, several opinion lexicons are publically available like SentiWordNet [3], General Inquirer [4], SenticNet [5], and so forth. But it is very difficult to construct a large opinion lexicon, which may have polarity of all the words, which may be used in any domain with accurate polarity because a word may be positive in one domain and the same words may have negative polarity in another domain, for example, sentence 1, “The story is unpredictable” and sentence 2, “The steering wheel is unpredictable.” In sentence 1, “unpredictable” word is opinion word, which is carrying a positive sentiment, and the same word “unpredictable” in sentence 2 is having negative sentiment. Opinion words change their polarity value depending on the context. Therefore, it is necessary to obtain the accurate polarity value of a word with the help of contextual information of opinion word [6, 7].

First important task in sentiment analysis is to identify the opinion targets (aspects, entities, and topic identification problems) about which some opinion is expressed. And, the second is to construct the opinion lexicon (good, excellent, etc.), for example, “I am very glad with the ambience of this restaurant.” “Ambience” is the opinion target of the writer and “glad” is the opinion word in this example. In the proposed work, overall polarity is computed at document level considering aspect level polarity of various aspects. Polarity of various aspects are computed and aggregated. The opinions of various aspects are based on the importance of various aspects with respect to the main domain, for example, “the audio quality of this phone is awesome but the pictures taken by its camera is not good.” In most of the existing sentiment analysis model, the above sentence may produce neutral or negative sentiment. But, for most of the people audio quality of the phone is more important than picture quality of the phone. Therefore, overall review polarity should be positive. Proposed model would be able to incorporate the importance of the features into consideration in determining the overall sentiment of the document.

Common-sense reasoning is often performed through common-sense ontologies and the use of reasoning algorithms, such as predicate logic and machine learning, to reach a conclusion [8]. Concept-level text analysis focuses on a semantic analysis of text through the use of web ontologies or semantic networks, which allow the aggregation of conceptual and affective information associated with natural language opinions [9, 10]. The main contribution of this paper is to propose a system, which selects only important features and aspects about which any opinion is expressed with the help of automatically constructed ontology. With the help of the domain specific ontology consisting of the common-sense knowledge produces only useful features. Further, polarity of an opinion word is determined with the help of contextualized sentiment lexicon.

In this paper, we propose an approach, which selects only important features and aspects about which any opinion is expressed with the help of automatically constructed ontology containing domain specific common-sense knowledge. Further, only domain specific concepts are extracted with the help of this ontology. Further, polarity of an opinion word is determined with the help of contextual sentiment lexicon. Further, sentiment information with respect to each attribute of the product is aggregated according to its importance with the topic, and finally sentiment of the document is determined, that is, positive or negative.

## 2. ConceptNet

ConceptNet is a large semantic network consisting of large number of common-sense concepts [11, 12]. Common-sense knowledge in ConceptNet is contributed by ordinary people on the Internet. It is the largest machine usable common-sense resource consisting of more than 250,000 relations. It is the largest publicly available common-sense knowledge base which can be used to mine various inferences from the text. It consists of nodes (concepts) connected by edges (relations between concepts). Some of the relationships between concepts in the ConceptNet are IsA, EffectOf, CapableOf, MadeOf, DesireOf, and so forth [12]. In ConceptNet, an assertion is defined by five properties, that is, language, relation, concept 1, concept 2, and frequency. Here, concept 1 and concept 2 are the two concepts which are having a relation. Language property defines the language of the assertion (in our case English). Frequency property represents how often given concepts are used with the given relation, for example, ConceptNet relations, Restaurant UsedFor eat, Restaurant IsA place, and so forth.

**Figure 1:**
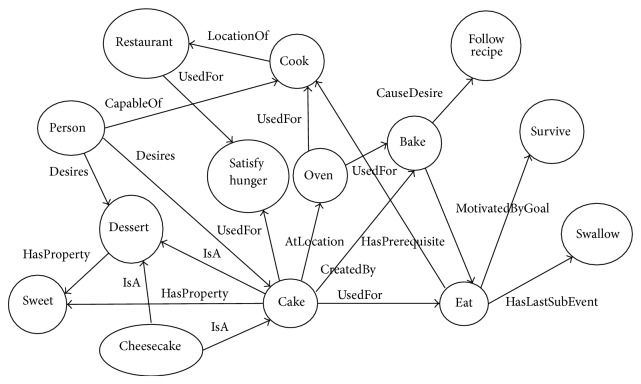
Sample ConceptNet ontology.

The ConceptNet semantic graph represents the information from the Open Mind corpus as a directed graph, in which the nodes are concepts and the labeled edges are common-sense assertions that interconnect them. For example, given the two concepts “person” and “cook,” an assertion between them is CapableOf; that is, a person is capable of cooking, as shown in Figure 1 [12].

## 3. Related Work

Techniques employed by sentiment analysis models can be broadly categorized into machine learning [13] and semantic orientation approaches [14]. Further, two types of techniques have been described in the literature for semantic orientation based approach for sentiment analysis, namely, corpus based and dictionary based. In corpus based approach, polarity value is computed based on the cooccurrences of the term with other positive or negative seed words in the corpus; there are various methods reported in the literature to determine the polarity value of the term, whereas, dictionary based approaches utilize the predeveloped polarity lexicons like SentiWordNet [3], WordNet, SenticNet [5], and so forth. These methods are also called as lexicon or knowledge based approaches. Semantic orientation based approaches for sentiment analysis work in the following phases. Initially, sentiment-rich features are extracted from the unstructured text. Further, semantic orientations of these sentiment-rich features are determined based on corpus or dictionary. Finally, overall polarity of the document is determined by aggregating the semantic orientations of all the features.

Automatic opinion target identification in the given sentence is very important subproblem of sentiment analysis. Opinion target extraction is more important in product reviews to identify the product features because each product may have large number of features and it is very difficult to make an explicit list of such features for every product. Therefore, it is necessary to have such mechanism, which may automatically identify the product features from the text. Ferreira et al. [15] present a comparative study of product feature extraction algorithm for customer reviews. Qiu et al. [16] proposed a semisupervised double propagation approach for the opinion lexicon expansion and target extraction problems simultaneously.

Feature-specific sentiment analysis finds out the opinion expressed with respect to a specific feature of the product. Several approaches have been proposed to deal with this problem [6, 16, 17]. Hu and Liu [17] proposed unsupervised model for feature-specific sentiment analysis; they discovered the product features using the association rule mining, considering frequent noun and noun phrases from the documents. Further, Wei et al. [18] improved their approach by considering two limitations in their method, namely, “frequent but nonproduct features” and “infrequent but product features” by semantic-based refinement of the frequent features obtained through the association mining approach that leverage the subjective adjectives from general inquirer. To determine the accurate polarity of the features is the key phase for building efficient sentiment analysis model. Most of the sentiment analysis models use domain-independent sentiment dictionary to determine the polarity of the features. The first work in determining semantic orientation of the adjective words was proposed by Hatzivassiloglou and McKeown [19]. Esuli and Sebastiani [3] proposed a method to determine the semantic orientation of subjective words based on quantitative analysis of the glosses of these words. Turney and Littman [20] introduce the method for inferring the semantic orientation of a word based on the statistical association of a word with the fixed set of positive and negative words. They experimented it with two approaches, namely, point-wise mutual information (PMI) and latent semantic analysis (LSA). Kamps et al. [21] proposed a method for determining the polarity of a word by its shortest paths to two seed words “good” and “bad” in WordNet. However, the main problem with most of the existing methods is that they are unable to incorporate the contextual information. In the literature, varieties of sentiment lexicons are freely available, but those lexicons do not provide contextual information in determining polarity value of a feature. Most of the sentiment lexicons available publically like SentiWordNet, General Inquirer, SenticNet, and so forth are domain-independent and sentiment analysis problem is domain dependent problem. Semantic orientation of a word changes according to the domain or the context in which that word is being used. For example, “unpredictable” is often used as a positive sentiment word in “movie” domain as in “unpredictable plot,” whereas the same word may have negative sentiment in “car” domain as “unpredictable steering.” In the proposed approach, we combined various sentiment lexicons present in the literature and also build contextualized sentiment lexicon. Several techniques have been proposed to include the contextual information in determining the polarity value of words. Wilson et al. [22] proposed a new approach to automatically determine the contextual polarity of sentiment expressions. Sharma et al. [23] proposed a method to construct the domain specific sentiment lexicon that incorporates the contextual information in determining the semantic orientation of words. Chi-square test is used to detect the words, which change the polarity depending on the domain.

ConceptNet as a knowledge resource is also used for sentiment analysis [24]. Sureka et al. [24] developed domain specific ontology from ConceptNet for target specific sentiment analysis. Mukherjee and Joshi [25] proposed a method for sentiment aggregation using domain specific ontology developed using ConceptNet. Our work is different from these approaches in the sense that we expanded the ontology with the help of WordNet for better coverage of the product features and also we used contextual polarity lexicon developed by us to determine the polarity of the extracted features.

## 4. Proposed Methodology

**Figure 2:**
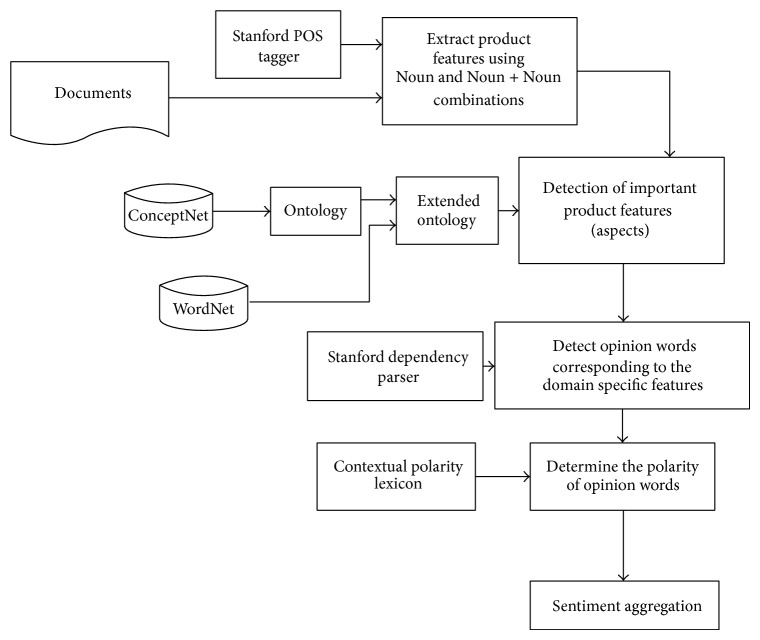
Flow diagram of proposed approach based on common-sense and context information.

In the proposed approach, initially, we use ConceptNet to construct a domain specific ontology for product reviews. Further, WordNet is used to expand the ontology for better coverage of the product features. Next, product features aspects/entities are identified using the ontology developed in the initial phase, for example, “battery life,” “image quality,” and “resolution” in the camera domain and “service,” “ambience,” “price,” and so forth in restaurant domain. Next, opinion or sentiments expressing phrases are extracted, for example, “extremely comfortable,” “good,” “bad,” and so forth. Further, semantic orientations of these phrases are determined corresponding to the entities extracted in the first phase with the help of contextual sentiment lexicon. Finally, overall sentiment orientation of the document is determined by aggregating the polarity values of all the phrases corresponding to all the entities.  Figure 2 presents the flow diagram of the proposed approach.

### 4.1. Construction of Domain Specific Ontology from ConceptNet

Ontology can be described as a connection of concepts with semantic relations. Semantic relations among concepts can be useful in inferring important information from the text. Constructed ontology can be considered as a common-sense knowledge base consisting of the domain specific concepts and relation among them.

**Figure 3:**
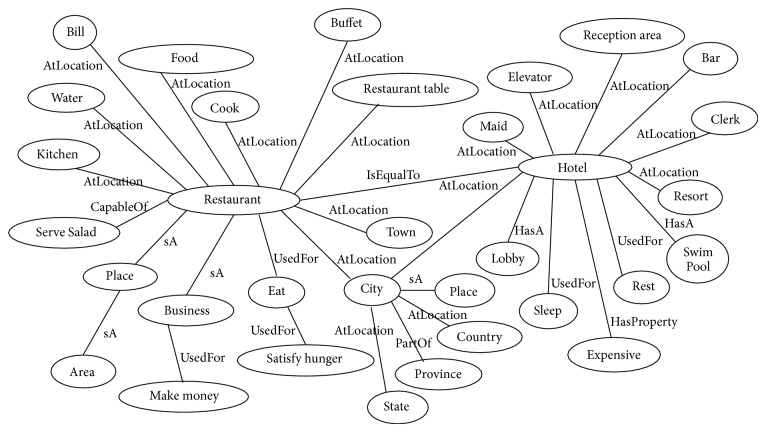
Sample ontology for restaurant domain.

In the proposed approach, initially, we construct the domain specific ontology with the help of ConceptNet. Next, we expand the ontology by merging the ontologies of the synonyms of the domain name for better coverage of domain specific features. For example, to build the ontology for restaurant, we also construct ontology for “hotel” and further merge both ontologies by connecting them with a new relation (i.e., IsEqualTo). The process of constructing the ontology is described in Algorithm 1. We extract the concepts from the ConceptNet upto level 4. The level of the ontology is set empirically. It is observed that as we increase the levels in ConceptNet, irrelevant concepts are extracted.  Figure 3, demonstrates the sample ontology for “restaurant” domain.

### 4.2. Aspect Extraction

Aspect is a term about which an opinion is expressed. In this step, we detect the aspects in a given sentence [26], for example, “this is a nice phone.” Here, “phone” is an aspect about which sentiment is expressed. To identify the aspect term, first of all, reviews are part-of-speech tagged and all the noun and noun + noun terms are extracted [17]. Stanford POS-tagger is used for tagging the review documents. For the same example, part-of-speech tagged sentence is as follows: “this DT is VBZ a DT nice JJ phone NN.” Here, phone is an aspect. Another example is as follows: “it PRP has VBZ long JJ battery NN life NN.” Here, battery life is considered as an aspect due to noun + noun rule. After the extraction of noun and noun + noun based aspects, these are matched with the domain specific ontology constructed in earlier section to eliminate all the unimportant and irrelevant aspects. All the irrelevant nouns-based aspects are pruned with the help of ontology.

### 4.3. Feature-Specific Opinion Extraction

Users generally express opinion on multiple features of product. User may have different opinion with respect to each feature in the review document, for example, “battery life of this phone is long, but appearance is bad.” Here, opinion towards “battery life” is positive and it is negative towards “appearance.” It is important to get the association of the opinion target (i.e., aspect) and opinion words [16, 27]. In this example, “battery life” is an aspect and “long” is an opinion word. To detect the association between opinion target and opinion words dependency parsing is used [25]. Stanford dependency parser is used for extracting dependency rules from the review documents.

### 4.4. Construction of Contextual Polarity Lexicon

**Figure 4:**
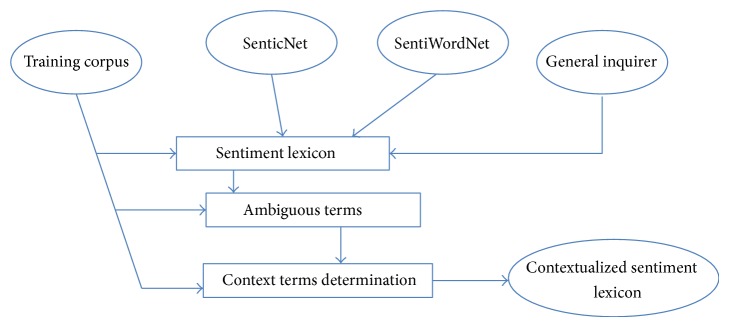
Flow diagram of construction of contextual polarity lexicon.

A polarity lexicon is a dictionary containing the terms/words with their polarity value. We build the sentiment lexicon with the help of three publically available resources, namely, SenticNet, SentiWordNet, and General Inquirer. Further, we determine the ambiguous terms from this sentiment lexicon and determine their polarity depending on the context in which they appear. Finally, polarity of the opinion word is retrieved from sentiment lexicon and contextual polarity lexicon. The process of the construction of contextual polarity lexicon is demonstrated in Figure 4.

*(1) SenticNet*. SenticNet is a publicly available resource for sentiment analysis. It is a lexical resource constructed by clustering the vector space model of affective common-sense knowledge extracted from ConceptNet. This dictionary produces a list of concepts with their polarity value. Polarity value of the concepts given in this dictionary is computed by exploiting artificial intelligence (AI) and semantic web techniques. It contains more than 14000 concepts along with their polarity scores. Polarity scores are in the range from −1.0 to +1.0. These concepts are a combination of single word and multiword concepts, having 7600 multiword concepts.

*(2) SentiWordNet*. SentiWordNet is a sentiment dictionary containing polarity score of opinion words [3]. SentiWordnet contains polarity of around 2 million words for adjectives, adverbs, verbs, and nouns part-of-speech tagged words. Words in SentiWordNet are divided in four categories: adjective, adverb, verb, and noun. It can be obtained from (http://sentiwordnet.isti.cnr.it). SentiWordNet was built using WordNet and a ternary classifier. The classifier is based on “bag of synset” model which uses manually disambiguated glosses available from the Princeton WordNet Gloss Corpus. It is a WordNet like lexicon which contain words with three scores as given below, that is,

- positive score,
- negative score,
- objective score.

For every word, positive, negative and neutral scores are having values between 0.0 and 1.0 and the addition of all the scores, that is, positive score, negative score, and objective score for a word, is 1. The objective score of 1.0 denotes that it is a neutral word and does not express any opinion.

*(3) General Inquirer*. The General Inquirer (GI) was one of the first sentiment dictionaries, publicly available dictionary, used for automatic text analysis [4]. It is a dictionary containing the positive and negative words. We combined all the three lexicons, and prepared a sentiment lexicon, which contains words with their polarity scores.

#### 4.4.1. Ambiguous Term Determination

The words, which change their polarity depending on the context, are called ambiguous words [28], for example, “I have a small complaint against this room is that tap is not working properly in the bathroom.” In this example, “complaint” is a term with negative sentiment but its polarity is changed to neutral with the context term small. The terms, which occur dominantly in positive context and occur very less in negative context, are most likely to be unambiguous terms. In contrast, the terms, which occur equally in positive and negative contexts may be ambiguous terms. We use Algorithm 2 to detect such ambiguous terms from the polarity lexicon.

Initially, we compute the mean sentiment score (*μ*) and standard deviation score (*σ*) of all the sentiment terms from the polarity lexicon. Mean sentiment and standard deviation scores are computed on the basis of distribution of occurrences of these terms in the training review documents. Further, the terms, which have high standard deviation score, are considered to be ambiguous terms. The threshold values for mean sentiment score and standard deviation score were determined empirically (as demonstrated in Algorithm 2). All the terms, which are not ambiguous, are considered as pure polar terms; that is, they generally do not change polarity with the context.

#### 4.4.2. Context Term Determination

Context terms are cooccurring terms with the ambiguous terms, which can change the polarity of the term [22]. We need to consider the context term to determine the accurate polarity of the ambiguous terms. In this step, we find the context terms corresponding to each ambiguous term detected in the previous step. And further these context terms are used to determine the polarity of the ambiguous term. Context terms are considered as all the nouns, adjectives, adverbs, and verbs that occurred in two sentences before and after the occurrence of the ambiguous term in all the review documents [22]. Further, for each context term, we compute the probability belonging to positive sentiment class and negative sentiment class. Positive and negative class probability is computed as follows:

(1) $$\begin{array}{c} \text{Positive}\_\text{probability}=\frac{P}{\left(P+N\right)},\\ \text{Negative}\_\text{probability}=\frac{N}{\left(P+N\right)}. \end{array}$$

Here, *P* is the total count of the term in positive training documents and *N* is the total count of the term in negative training documents. If the context term has significantly higher positive class probability, then that term contributes more towards positive polarity and vice versa. Therefore, we consider a context term as positive if it has a higher positive probability and consider it negative otherwise. We sum up the polarities of all the context terms to determine the polarity of the ambiguous term. Finally, we compute the polarity for all the ambiguous terms determined in the previous steps.

### 4.5. Sentiment Aggregation

(1) First of all, features are extracted from the review document, and then it is matched in the ontology. The level of ontology where it is located determines the importance of the feature. The features located at higher level near to the root of the ontology are considered to be more important as compared to the lower level features. Further, opinion word corresponding to this feature is detected using dependency parsing rules.

(2) Further, the polarity of the opinion word is determined as follows. Initially, polarity of an opinion word is retrieved from ambiguous polarity list, if the word is present. If the opinion word is not ambiguous, then polarity value is retrieved from unambiguous polarity lexicon (i.e., Combination of SenticNet, SentiWordNet, and General Inquirer). Contribution of the opinion word in determining the overall polarity is taken as polarity∗(height of ontology). Finally, the overall polarity of the document is determined by summing up the contribution of each term.

## 5. Experiments and Results

### 5.1. Dataset and Evaluation

To evaluate the performance of the proposed methods, three standard benchmark datasets are used. First is the restaurant review dataset available at http://people.csail.mit.edu/bsnyder/naacl07/data/unformatted/. This corpus consists of 4,488 reviews. Polarity of the review documents is classified as positive or negative. Second corpus is the movie review dataset provided by Pang and Lee [29]; it consists of 2000 reviews of equal number of negative and positive reviews. And the third dataset is the software review dataset, provided by Blitzer et al. [30]. It consists of 1000 positive and 915 negative review documents. For the evaluation of proposed methods, we randomly divide the dataset into 90% training and 10% testing documents, so that both sets are disjoint. We repeat all the experiments 10 times, and final performance is reported by averaging the results. Accuracy is used as an evaluation measure. It is computed by dividing the total correctly classified testing documents by the total number of testing documents. All the features/words extracted from the review documents are lemmatized to reduce to their root form for better matching of features in the ontology. A simple negation handling method is used in all the experiments. In this method, negation terms (no, not, and never) are added to all the adjectives present in the sentence to reverse the polarity of each term. For example, “this is not a good movie,” is converted to “this is a not good movie.”

### 5.2. Experiments

There are three objectives of this paper. First is to investigate the effectiveness of the ontology used to prune the irrelevant features extracted from the review documents. It is done by selecting only domain specific important features based on ConceptNet. Second is to investigate the effectiveness of incorporating the importance of the features in determining the overall sentiment of the document. Third is to evaluate the contextual polarity lexicon in determining the polarity of the opinion words.

#### 5.2.1. Baseline

A simple lexical based approach is considered as baseline in our experiments [25, 28, 31]. In this approach, a sentiment lexicon is taken to retrieve the polarity value of all the features extracted from the review document. Then, it sums up the total positive and negative polarity values of all the words of the document; if total positive polarity is greater than total negative polarity value, then the positive sentiment was assigned to document and vice versa.

#### 5.2.2. Domain Specific Ontology Based Method

In this experiment, we evaluate the contribution of the domain specific ontology in improving the performance of sentiment analysis. In this experimental setting, we extracted the features with noun and (noun + noun) combinations from the review documents; further, extracted features are matched in the ontology to select only domain specific important features. Then, dependency parsing is used to get the opinion words corresponding to the features extracted in first phase. Then, all the three lexicons are used to get the polarity value of the opinion words. Finally, polarity value of all the opinion words in a document is summed to get the final polarity of the document.

#### 5.2.3. Considering the Importance of the Features

This experiment is to investigate the effect of considering the importance of the feature in determining the overall sentiment of the document. This approach is similar to previous approach, that is, domain specific ontology based method except in polarity computation; we consider the importance of the feature by looking at the level of match of the feature in the domain specific ontology.

#### 5.2.4. Contextual Sentiment Lexicon

This experiment is to evaluate the performance of the sentiment analysis model when we consider the context information. In this experiment, we add the contextual polarity lexicon, in addition to all the three lexicons. All other settings are same as method 2.

#### 5.2.5. Considering Contextual Information and Importance of the Feature

In this experiment, we investigate the effect of the context information, importance of the features, and domain specific ontology, all together to determine the efficiency of the proposed sentiment analysis model.

### 5.3. Results

Table 1 presents the results of all the experiments with three standard datasets. The baseline method gives the accuracy of 65.7%, 67.8%, and 70.1% for restaurant review dataset. Next, the accuracy is improved with method 2 by incorporating domain specific ontology to get only domain related features as shown in Table 1. For example, accuracy is increased from 67.8% to 69.2% (+2.0%) for the software review dataset. Further, method 3 improves the efficiency of the sentiment analysis model by considering the importance of the features. Accuracy improves from 67.8% to 72.6 (+7.07%) for software review dataset. Both methods are unsupervised; we only require a predefined ontology and prebuilt polarity lexicons. It is observed from the experiments that the effect of common-sense knowledge based ontology slightly depends on the type of the dataset. It improves the performance for the domains, which have more possible aspects. For example, restaurant and software domain reviews have more possible aspects as compared to movie review dataset.

Further, by considering the context information in determining the polarity of the opinion words, performance is improved for all the datasets. For example, accuracy is increased from 67.8% to 77.3% (+14.01%) for software review dataset. Finally, performance is significantly increased from 67.8% to 80.1% (+18.14%) for software domain when considering all the information, that is, common-sense knowledge based domain specific ontology, importance of the extracted feature, and contextual information.

### 5.4. Comparison with Related Work

The use of ConceptNet for sentiment analysis has not been explored much in the literature. Our proposed approach is quite similar to the approach proposed by Sureka et al. (2013). They developed domain specific ontology from ConceptNet for target specific sentiment analysis. Mukherjee and Joshi [25] proposed a method for sentiment aggregation using domain specific ontology, which was developed using ConceptNet. Our work is different from these approaches in the sense that we expanded the ontology with the help of WordNet for better coverage of the product features and also we used contextual polarity lexicon developed by us to determine the polarity of the extracted features. The proposed approach produces the best accuracy of 80.1% on software review dataset, and Mukherjee and Joshi [25] give the best accuracy of 76.06% on the same dataset.

## 6. Conclusions

In this paper, we investigate the effect of three factors, that is, domain specific ontology, importance of the features, and contextual information all together in determining the overall sentiment of text. The proposed approach including all these information significantly improves the performance of the sentiment analysis model over the baseline method. All the experiments are performed on various review datasets, namely, software, movie, and restaurants. Experimental results show the effectiveness of the proposed approach. Future work involves discovering methods to enrich the knowledge base. Along with using ConceptNet, how other ontologies can help to enrich the concept mining process is also a big task to deal with in future.

## Figures and Tables

**Algorithm 1:**
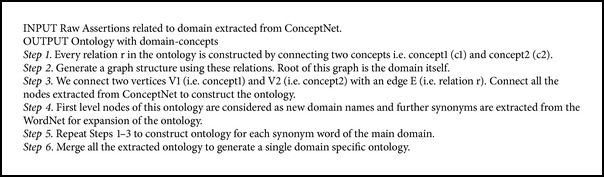
Build domain specific ontology from common-sense knowledge base.

**Algorithm 2:**
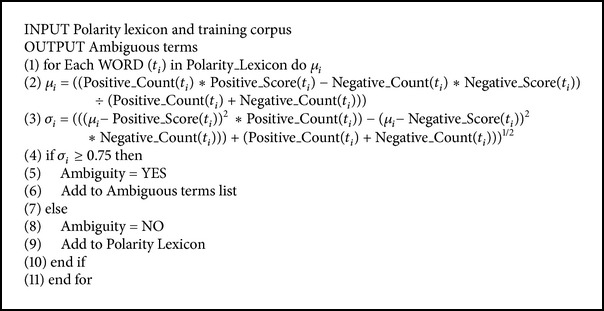
Finding ambiguous terms.

**Table 1:** Accuracy (In %) of various methods on different datasets.

Method	Software	Movie	Restaurant
Method 1 (baseline)	67.8	70.1	65.7
Method 2 (with domain specific ontology)	69.2 (+2.0%)	71.3 (1.2%)	68.3 (+3.9%)
Method 3 (considering importance of the feature)	72.6 (7.07%)	71.9 (+2.5%)	71.1 (+8.2%)
Method 4 (with contextual information)	77.3 (+14.01%)	76.2 (+6.1%)	76.2 (+15.9%)
Method 5 (with context information and importance of the feature)	80.1 (+18.14%)	78.9 (+12.5%)	79.4 (+20.8%)
